# Silencing CDK4 radiosensitizes breast cancer cells by promoting apoptosis

**DOI:** 10.1186/1747-1028-8-10

**Published:** 2013-07-25

**Authors:** Katie R Hagen, Xiangbin Zeng, Mi-Young Lee, Shannon Tucker Kahn, Mary Kathryn Harrison Pitner, Sandra S Zaky, Yuan Liu, Ruth M O’Regan, Xingming Deng, Harold I Saavedra

**Affiliations:** 1 Department of Radiation Oncology, Emory University School of Medicine, Atlanta, USA; 2 Department of Medical Oncology, Emory University School of Medicine, Atlanta, USA; 3 Department of Biostatistics and Bioinformatics, Emory University School of Public Health, Atlanta, USA

**Keywords:** CDK4, CDK2, Radiation, Bad, Radioresistance, Radiosensitization, Breast cancer, Apoptosis

## Abstract

**Background:**

The discovery of molecular markers associated with various breast cancer subtypes has greatly improved the treatment and outcome of breast cancer patients. Unfortunately, breast cancer cells acquire resistance to various therapies. Mounting evidence suggests that resistance is rooted in the deregulation of the G1 phase regulatory machinery.

**Methods:**

To address whether deregulation of the G1 phase regulatory machinery contributes to radiotherapy resistance, the MCF10A immortalized human mammary epithelial cell line, ER-PR-Her2+ and ER-PR-Her2- breast cancer cell lines were irradiated. Colony formation assays measured radioresistance, while immunocytochemistry, Western blots, and flow cytometry measured the cell cycle, DNA replication, mitosis, apoptosis, and DNA breaks.

**Results:**

Molecular markers common to all cell lines were overexpressed, including cyclin A1 and cyclin D1, which impinge on CDK2 and CDK4 activities, respectively. We addressed their potential role in radioresistance by generating cell lines stably expressing small hairpin RNAs (shRNA) against CDK2 and CDK4. None of the cell lines knocked down for CDK2 displayed radiosensitization. In contrast, all cell lines knocked down for CDK4 were significantly radiosensitized, and a CDK4/CDK6 inhibitor sensitized MDA-MB-468 to radiation induced apoptosis. Our data showed that silencing CDK4 significantly increases radiation induced cell apoptosis in cell lines without significantly altering cell cycle progression, or DNA repair after irradiation. Our results indicate lower levels of phospho-Bad at ser136 upon CDK4 silencing and ionizing radiation, which has been shown to signal apoptosis.

**Conclusion:**

Based on our data we conclude that knockdown of CDK4 activity sensitizes breast cancer cells to radiation by activating apoptosis pathways.

## Background

Breast cancer is the most common malignancy and the major cause of cancer related deaths of women in industrialized countries [1]. Fortunately, the discoveries of various biomarkers have greatly improved the treatment of breast cancer patients [2]. For example, breast cancer patients that express progesterone receptor (PR) and estrogen receptor (ER) (luminal A breast cancers are ER+PR+HER2- or ER+PR+HER2+, while luminal B are ER+PR+HER2-) are treated with endocrine therapies and/or with the antibody Herceptin, in combination with chemotherapy and/or radiotherapy [3]. Regrettably, the defining characteristic of triple negative breast cancers (a subtype of basal breast cancers) is the absence of ER, PR and HER2, rendering those cancers insensitive to some of the most effective therapies [4]. ER-PR-HER2- and ER-PR-HER2+ patients show decreased survival relative to luminal subtypes [5,6].

Radiotherapy is one of the cornerstones in the treatment of patients with breast cancer [7,8]. Radiation greatly reduces the risk of recurrence in women with ductal carcinoma *in situ*[9,10] and in breast cancer patients who are lymph node positive [11-13]. The outcomes following chemotherapy vary depending on the subtype: for example, ER-PR-HER2- and ER-PR-HER2+ breast cancers respond better than luminal subtypes to anthracycline-based chemotherapies [14]. In contrast, all subtypes respond equally well to radiation [15]. Regardless of treatment, ER-PR-HER2- or ER-PR-HER2+ breast cancers are more likely to recur upon breast conserving therapies or following mastectomy and radiation [16-19]. Even though radiotherapy shows promise in the treatment of all breast cancer subtypes, radiotherapy is associated with increased risk of cardiovascular disease [20]. In addition, breast tumors can acquire radioresistance [21,22]. Finding agents that sensitize malignant cells to radiation would increase tumor response while minimizing toxicity to surrounding organs by lowering effective therapeutic doses.

The G1/S phase regulatory machinery includes cyclins and cyclin kinase inhibitors (CKIs) that regulate the activities of the G1 phase CDKs [23,24] and the Rb/E2F pathway [25,26]. Besides being distinguished by their ER, PR, or Her2 status, the various breast cancer subtypes exhibit deregulated expression of proteins that ensure progression through the G1/S phase of the cell cycle. ER-PR-HERr2- breast cancers are more likely to overexpress mutant p53, E2F3, p16, and cyclin E and display lower levels of cyclin D1, Bcl2, and Rb relative to other breast cancer subtypes [5,6,27,28]. Cyclin D1 overexpression is more often found in tumors with wild type p53, higher grades of differentiation, and expression of ER or PR [29-32]. Specifically, 53% PR+ and 58% ER+ breast cancer patients overexpress cyclin D1, while a smaller, but significant fraction of ER- and PR- breast cancers (21% and 31% respectively) overexpress cyclin D1 [33].

Several studies have shown that G1/S phase regulatory molecules may drive lower survival rates in patients and resistance to adjuvant therapies. Deregulated expression of cell cycle molecules that specifically modulate CDK2 kinase activity has been associated with poor prognosis of breast cancer patients. For example, high cyclin A expression in metastatic breast cancer correlates significantly with a shorter time to first relapse and is a prognostic factor in early stage ER+ breast tumors [34,35]. Additionally, high cyclin E expression predicts a poor prognosis in breast cancer [36,37]. Unregulated CDK2 activity may result in poorer survival due to the modulation of responses to various therapeutic agents. For example, low expression of p27^Kip1^ and/or cyclin E overexpression predicts early relapse in patients treated with adjuvant therapy that includes tamoxifen [38,39], doxorubicin, cyclophosphamide [40], and Herceptin [41]. Increased CDK2 kinase activity drives Herceptin resistance *in vitro* and *in vivo*[41,42].

Deregulated CDK4/CDK6 activities have also been associated with decreased survival and resistance to various therapies. Cyclin D1 is overexpressed in over 50% of breast cancers [43,44]. The oncogenic capacity of cyclin D1/CDK4/CDK6 in experimental models, including mouse models of mammary carcinogenesis has been established [45-47]. However, the role of cyclin D1/CDK4/CDK6 in breast cancers is highly controversial. For example, increased cyclin D1 mRNA and amplification of *CCND1* in ER+ breast cancers strongly correlated with increased risk of relapse, local recurrence, metastasis, and death [30,31,48], and ER- patients with cyclin D1 overexpression display shorter overall survival [33]. In contrast, other studies showed that patients overexpressing cyclin D1 (including ER+ tumors and unclassified breast tumors not falling within the canonical HER2, PR, ER classification) were less likely to recur following treatment and displayed longer survival [32,49-51]. Similar to cyclins E and A, there is experimental evidence as to the involvement of cyclin D in resistance or sensitivity to various therapies. Patients within the ER+ subgroup who received endocrine therapy for their primary or recurrent breast cancers showed an association between high cyclin D1 and a shorter response duration [30,52,53]. In addition, *in vitro* studies showed that overexpressed cyclin D1 leads to resistance to antiestrogens [54]. While some studies showed that ectopic cyclin D1 expression is directly involved in radioresistance and the poor prognosis of various carcinomas after radiotherapy [55-59], others found that ectopic expression of cyclin D1 markedly increases cell sensitivity to apoptosis induced by various agents including ionizing radiation [60-63]. In addition, chemical inhibition of CDK4 and CDK6 synergizes with Herceptin and tamoxifen treatments [64].

Although CDK4 and CDK2 are promising targets in cancer therapeutics, their role in the response of ER-PR-HER2+ or ER-PR-HER2- breast cancer cells to ionizing radiation is controversial and not extensively explored. We present data showing that knockdown of CDK4, but not of CDK2, imparts radiosensitivity to breast cancer cells and normal mammary epithelial cells by signaling an apoptotic program.

## Results

### G1/S phase regulatory molecules are ectopically expressed in radioresistant breast cancer cells

While many deregulated signaling pathways, including PI3K, NFKB, and the MAPK pathways are involved in signaling radiation resistance [65], the role played by the G1/S phase regulatory machinery phase in radiation resistance is unclear. The overall hypothesis of this study is that breast cancer cells are radioresistant because they harbor deregulated G1/S phase cell cycle machinery. The controversy regarding the role played by the G1/S phase regulatory machinery in radioresistance may be due to the use of normal or transformed cells lines, their tissue of origin, or to the dose and duration of radiation treatments [55,58-60]. Because of this, we tested our hypothesis in a non-transformed mammary epithelial cell line and breast cancer cell lines of various molecular subtypes, irradiated with increasing, single doses of radiation. We first assessed the ability of MCF10A (non-transformed, immortalized mammary epithelial cells lacking the cyclin kinase inhibitors p16^INK4A^ and p15^INK4B^ due to a homozygous deletion), ER-PR-HER2- and ER-PR-HER2+ breast cancer cells to form colonies after increasing single doses of γ-radiation (0, 1, 2, 4, 6, or 8 Gy). Analyses revealed that most of the breast cancer cell lines tested in this study showed resistance to radiation with a statistically significantly increased IC_50_ comparing to the immortalized MCF10A cells (Table 1).

**Table 1:** **IC**_**50 **_**of Irradiation in non-transfected human breast cell lines**

**Cell line**	**Cell type**	**IC**_**50**_	
		**Est. (SE)**	**P value**^**1**^
MCF10A	Normal	1.54 (0.116)	-
MDA-MB-231	ER-PR- HER2-	1.93 (0.191)	0.04
HCC1806	ER-PR- HER2-	1.68 (0.128)	0.398
MDA-MB-468	ER-PR- HER2-	2.06 (0.155)	0.02
HCC1954	ER-PR- HER2+	2.60 (0.189)	<0.001
SKBR3	ER-PR- HER2+	2.08 (0.156)	0.001

^1^The p value is based on the comparisons relative to MCF10A group for each cell line.

**Figure 1:**
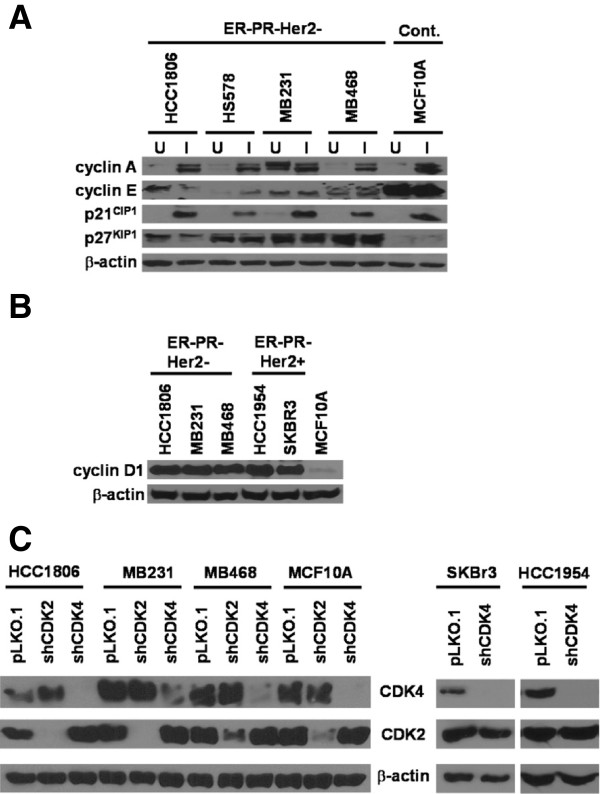
**G1/S phase regulatory molecules are ectopically expressed in radioresistant breast cancer cells. (A)** Non-transformed human mammary epithelial cells (MCF10A) and ER-PR-HER2- human breast cancer cell lines (HCC1806, HS578, MDA-MB-231 & MDA-MB-468) were irradiated at 2 Gy. Western blots of lysates prepared from both the unirradiated (U) and the irradiated (I) cells were immunoblotted with anti-cyclin A, anti-cyclin E, anti-p21^CIP1^ and anti-p27^KIP1^ antibodies. **(B)** Western blot analyses of lysates prepared from various unirradiated human breast cancer cell lines (triple negative and ER-PR-HeR2+ and MCF10A) were immunoblotted with anti-cyclin D1 antibody. **(C)** Lentivirus containing shRNA constructs specific for CDK2 or CDK4 were used to infect human breast cancer cell lines and MCF10A cells. A pLKO.1 lentiviral vector containing a randomized shRNA sequence was used as control. After puromycin selection, the surviving cells were pooled. The lysates prepared from the infected cells were immunoblotted with anti-CDK2 or anti-CDK4 antibodies to confirm the knockdown efficiency. Equal loading was assessed by immunoblotting with a β-actin antibody.

To identify mechanisms resulting in radioresistance, we assessed the relative protein expression of G1/S cell cycle regulatory molecules that have been shown to associate with relapse and to impaired responses to various therapies. We focused on proteins that affect CDK2 or CDK4 activities, including cyclins A, E, D, p21^CIP1^, and p27^KIP1^ (Figure 1). Western blot analyses showed that p27^KIP1^ levels were higher in untreated ER-PR-HER2- breast cancer cells relative to MCF10A cells and showed no change upon irradiation (Figure 1A). Levels of cyclin E were higher in MCF10A cells relative to breast cancer cells irrespective of irradiation. Other molecules, including cyclin A and p21^CIP1^, were upregulated in both ER-PR-HER2- and MCF10A cells after irradiation, while cyclin D1 levels did not change. We measured cyclin D1 levels in non-irradiated MCF10A, ER-PR-HER2-, and ER-PR- HER2+ cell lines and showed that all breast cancer cell lines overexpressed cyclin D1 (Figure 1B).

Because the breast cancer cell lines overexpressed cyclin D1, and cyclin A protein levels were elevated following irradiation, we tested whether knockdowns of their respective kinase partners CDK4 and CDK2 might alter the relative radioresistance of various breast cancer cell lines. Therefore, we generated ER-PR-Her2- and MCF10A cell lines stably expressing shCDK2 or shCDK4. All cell lines showed consistently stable decreases in the protein level of CDK2 or CDK4 (Figure 1C). MCF10A and HCC1806 cells displayed complete downregulation of CDK4, while MDA-MB-468 and MDA-MB-231 cells showed a partial, but almost complete knockdown of CDK4. In addition, HCC1806 and MDA-MB-231 cells displayed complete knockdown of CDK2, while MCF10A and MDA-MB-468 cells displayed a partial, but significant knockdown of CDK2. Importantly, knockdown of CDK2 did not affect CDK4 levels and *vice versa*, demonstrating that specific knockdowns were achieved. To assess whether silencing of CDK4 altered radioresistance in other breast cancer subtypes, we generated HCC1954 and SKBR3 cells (both ER-PR-HER2+ cell lines) knocked down for CDK4 (Figure 1C). Similarly to ER-PR-HER2- and MCF10A cells, CDK2 levels in ER-PR-HER2+ cells were not affected by knockdown of CDK4.

### Downregulated CDK4 sensitizes breast cancer cells to radiation

**Figure 2:**
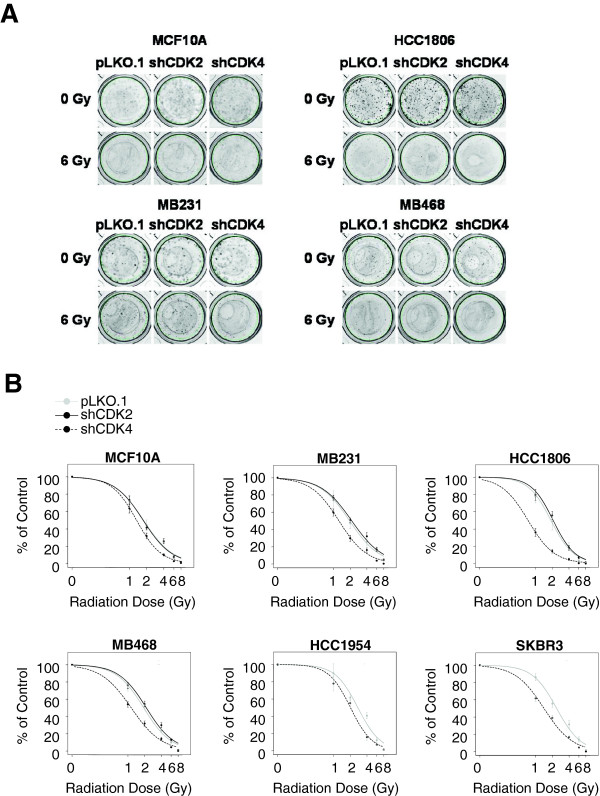
**Downregulated CDK4 sensitizes breast cancer cells to radiation.** Cells stably expressing pLKO.1, shCDK2 or shCDK4 were irradiated at single doses of 0, 1, 2, 4, 6, or 8 Gy γ-rays. After an average of 8 days, cells were fixed and stained with 1% crystal violet; colonies were counted manually. **(A)** The figure shows colony formation in MCF10A and ER-PR-HER2- cells at 0 and 6 Gy. **(B)** Survival curves of various cells transfected with pLKO.1, shCDK2 or shCDK4. ER-PR-Her2+cells were only transfected with pLKO.1 or shCDK4. The y axis represents percentage colony formation relative to unirradiated cells of the respective groups; the x axis represents the various radiation doses. The IC_50_ and statistical significance are presented in Table 2.

After cell lines stably silenced for CDK2 and CDK4 were established, all cell lines received ionizing radiation at doses ranging between 0 to 8 Gy. The relative radiosensitivity and the radiation dose that causes a 50% decrease in the number of colonies (IC_50_) were calculated using colony forming assays (Figure 2A, Table 2). Even though CDK2 has been reported to mediate resistance to various adjuvant therapies against breast cancers, downregulation of CDK2 did not alter the sensitivity (IC_50_) to radiation in ER-PR-HER2- cells or in MCF10A cells relative to cells expressing control vector (Figure 2B, Table 2). In contrast, all cell lines harboring CDK4 knockdown, including ER-PR-HER2+, ER-PR-HER2-, and MCF10A showed significantly decreased IC_50_, meaning that the ablation of CDK4 in both the normal and tumor cell lines sensitizes these cells to radiation.

**Table 2:** **IC**_**50**_**of irradiation in breast cancer cell lines with CDK2 or CDK4 knockdown**

**Cell line**	**Vector**	**IC**_**50**_	
		**Est. (SE)**	**P value**^**1**^
MCF10A	pLKO.1	1.76 (0.106)	-
	shCDK2	1.75 (0.103)	0.943
	shCDK4	1.34 (0.076)	0.005
MDA-MB-231	pLKO.1	1.89 (0.141)	-
	shCDK2	2.15 (0.158)	0.197
	shCDK4	1.27 (0.096)	0.003
HCC1806	pLKO.1	1.95 (0.149)	-
	shCDK2	2.14 (0.173)	0.364
	shCDK4	0.85 (0.078)	<0.001
MDA-MB-468	pLKO.1	2.04 (0.139)	-
	shCDK2	2.27 (0.156)	0.252
	shCDK4	1.25 (0.093)	<0.001
HCC1954	pLKO.1	2.99 (0.301)	-
	shCDK4	2.02 (0.160)	0.013

^1^The p value is based on the comparisons with pLKO.1 group within each cell line.

### Radiosensitivity imparted by downregulated CDK4 is not caused by cell cycle blocks

**Figure 3:**
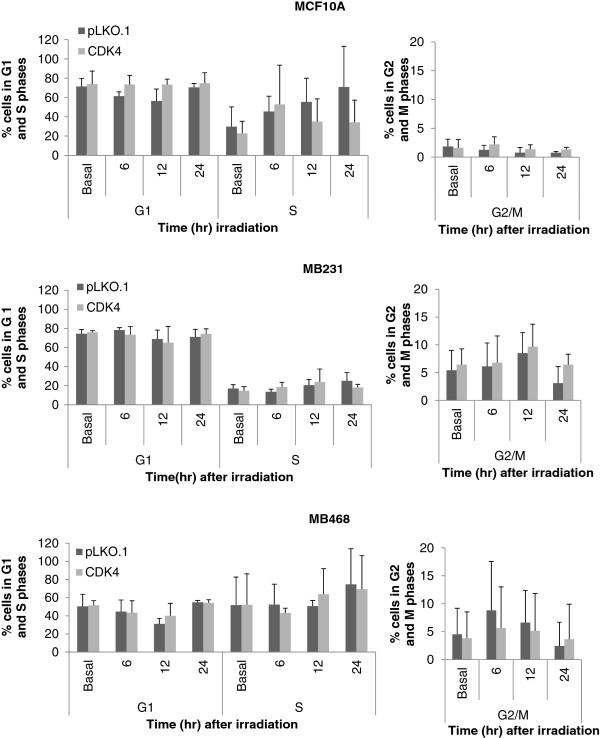
**Radiosensitivity imparted by downregulated CDK4 is not caused by altering cell cycle progression.** Cells stably expressing pLKO.1 or shCDK4 were unirradiated (basal), or irradiated at 2 Gy. Then, cells were collected at different times post-irradiation (0, 6, 12, & 24 hours) stained with 7-AAD and were analyzed using flow cytometry to determine the cell cycle stages; 5000 cells per group were analyzed by the flow cytometer. Flow cytometry and analysis was repeated three times. The columns represent the percentage of cells in the G1, S or G2/M phase and presented as average±standard deviation. The G1/S and G2/M graphs were separated in order to make the G2/M data visible.

Radioresistance can be a consequence of a deregulated cell cycle, altered frequencies of apoptosis, or altered frequencies of DNA repair [58-60]. To address whether radiosensitization occurs because of cell cycle blocks, we irradiated MCF10A and ER-PR-HER2- cells with 2 Gy and assessed percentages of proliferating cells using the BrdU incorporation assay by direct counting using fluorescence microscopy (Additional file 1A) and by flow cytometry (Table 3). Ki-67 was used as an independent marker of proliferation (Additional file 1B). Additional file 1 represents two preliminary experiments with a wider range of timepoints, to establish whether DNA replication was affected up to 48 hours post-irradiation. Neither BrdU nor Ki-67 immunocytochemistry showed differences in proliferation in cells downregulated for CDK4 relative to the control pLKO.1 group. Although cells expressing shCDK2 incorporated BrdU at a lower extent than other groups (Additional file 1A), no differences were found when using Ki67 as an independent marker of proliferation (Additional file 1B). After establishing that DNA replication was not affected, a smaller number of timepoints (0, 6, 12 and 24 hours post-irradiation) were chosen for final analyses. The relative percentage of cells in each phase of the cell cycle was assessed by measuring DNA content using flow cytometry (Figure 3), while a flow cytometry-based BrdU assay measured percentage of replicating cells post-irradiation (Table 3). The BrdU assay did not detect significant differences in the percentage of proliferating cells between control and cells silenced for CDK4 (Table 3). Likewise, flow cytometry did not reveal any major changes in the percentage of cells in G1, S, or G2/M phases of the cell cycle in irradiated or non-irradiated cells when comparing shCDK4 cells relative to pLKO.1 controls (Figure 3). Thus, downregulation of CDK4 does not radiosensitize cells through compromising their proliferation capacity.

**Table 3:** Comparison of BrdU between control (pLKO.1) and treatment (shCDK4) groups

**Cell line**	**Vector**	**6 hr**			**12 hr**			**24 hr**		
		Mean*	**std**	**P-value†**	**Mean**	**std**	**P-value†**	**Mean**	**std**	**P-value†**
MCF10A	pLKO.1	5.629	3.740	0.1124	2.669	0.956	0.3790	0.944	0.571	0.9577
	shCDK4	2.807	2.460		1.165	1.628		0.855	0.689	
MDA-MB-468	pLKO.1	1.619	0.614	0.7841	1.229	1.030	0.9942	6.503	7.950	0.0828
	shCDK4	0.842	0.915		1.208	0.946		1.254	1.685	
MDA-MB-231	pLKO.1	1.698	0.870	0.8941	1.578	1.634	0.1996	1.097	0.562	0.5523
	shCDK4	1.806	0.731		2.654	3.050		1.581	0.173	

* Mean (standard deviation) of the fold change to no radiation.

† P-value is for comparing in the mean fold change no radiation between control and treatment groups.

To measure if knockdown of CDK4 radiosensitizes cells by signaling mitotic blocks, cells were immunostained for phospho-histone 3 after irradiation. Histone 3 is one of eight core histone proteins that play an important role in transcription regulation in eukaryotes through various modifications, including phosphorylation [66]. Phosphorylation of histone 3 at Ser10, Ser28 and Thr11 is correlated with chromosome condensation during mitosis [67,68] and is regarded as an indicator of mitosis. Overall, no significant elevations in phospho-histone 3 positive cells were observed after irradiation in pLKO.1 or shCDK4 cells (Table 4). This demonstrates that CDK4 silencing does not radiosensitize cells by causing accumulation of cells in mitosis.

**Table 4:** Comparison of phospho-Histone 3 between control (pLKO.1) and treatment (shCDK4) groups

**Cell line**	**Vector**	**0 Gy**			**2 Gy**			**4 Gy**		
		Mean*	**std**	**P-value†**	**Mean**	**std**	**P-value†**	**Mean**	**std**	**P-value†**
MCF10A	pLKO.1	1.229	0.530	0.145	1.737	0.278	0.277	0.823	0.587	0.168
	shCDK4	2.001	0.961		1.560	0.385		1.498	0.891	
MDA-MB-468	pLKO.1	0.476	0.555	0.462	0.808	0.171	0.256	0.799	0.221	0.249
	shCDK4	0.509	0.077		0.654	0.330		0.979	0.359	
MDA-MB-231	pLKO.1	0.168	0.160	0.324	0.170	0.176	0.084	0.060	0.104	0.181
	shCDK4	0.342	0.593		0	0		0.257	0.315	

* Mean (standard deviation) of % of phospho-Histone 3 positive cells.

† P-value is for comparing in the mean between control and treatment groups in each irradiation dosage.

### Downregulation of CDK4 does not alter rates of DNA break repair

To establish whether increased DNA repair is responsible for radiation resistance, we measured DNA breaks by detecting γ-H2AX foci. H2A.X is a variant histone which is phosphorylated on Ser139 (γ-H2AX) by ATM, ATR, or DNA PK at sites of DNA damage to recruit DNA repair enzymes, thus representing an important mark of DNA breaks [69-71]. This mark is one of the most sensitive ways to detect active DNA repair foci [70,72]. Increased frequencies of γ-H2AX foci have been extensively used to measure DNA damage by physical agents, oncogenes, or inactive tumor suppressors [59,73,74]. Because phosphorylated γ-H2AX is a major sensor of double strand breaks, clearance of phospho-H2AX foci following irradiation is also used as a measure of the repair of that DNA damage [70,72]. Additional file 2 represents a preliminary experiment with a wider range of timepoints (up to 48 hours post-irradiation), to establish whether γ-H2AX foci were detected in non-irradiated cells (basal) or following irradiation with 2 Gy. To measure the relative repair capability of various cells, we irradiated cells with 2 Gy and detected γ-H2AX foci at different time points post-irradiation. γ-H2AX foci were clearly detectable upon irradiation (Additional file 2A). Regardless of the cell line, the percentage of γ-H2AX foci decreased gradually post-irradiation in all groups and reached basal levels by 24 hours. These baseline levels were maintained 48 hours post-irradiation. The γ-H2AX results obtained by immunocytochemistry were independently investigated in MCF10A, MDA-MB-231, and MDA-MB-468 cells using flow cytometry. This assay confirmed that no significant differences existed between control and cells silenced for CDK4 (Table 5). The experiments indicate that the double stranded DNA repair capacity of all cells analyzed in this study was intact.

**Table 5:** Comparison of γ-H2AX between control (pLKO.1) and treatment (shCDK4) groups

**Cell line**	**Vector**	**6 hr**			**12 hr**			**24 hr**		
		Mean*	**std**	**P-value†**	**Mean**	**std**	**P-value†**	**Mean**	**std**	**P-value†**
MCF10A	pLKO.1	3.964	1.839	0.4621	3.926	3.203	0.1512	1.308	0.357	3.964
	shCDK4	2.803	2.216		1.583	1.393		1.707	0.643	
MDA-MB-468	pLKO.1	2.148	1.176	0.8272	1.831	0.091	0.9269	4.835	5.890	0.1292
	shCDK4	1.660	2.104		1.626	1.407		1.273	0.729	
MDA-MB-231	pLKO.1	2.518	0.443	0.4119	3.016	1.634	0.6689	1.587	0.562	0.6609
	shCDK4	3.710	2.273		3.630	3.050		2.217	0.173	

* Mean (standard deviation) of the fold change to no radiation.

† P-value is for comparing in the mean fold change no radiation between control and treatment groups.

### Irradiated breast cancer cell lines silenced for CDK4 display increased frequencies of apoptosis

**Figure 4:**
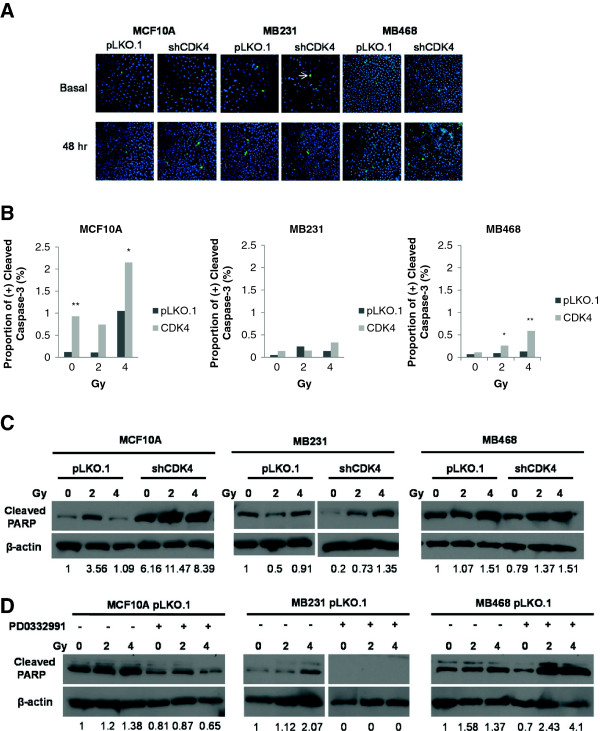
**Irradiated breast cancer cell lines knocked down for CDK4 display increased frequencies of apoptosis. (A)** Cells stably expressing pLKO.1, shCDK2 or shCDK4 were unirradiated (basal), irradiated at 2 or 4 Gy. Cells were fixed at 48 hours post-irradiation. Cells were subjected to immunostaining with anti-cleaved caspase-3 antibody (arrows), and an Alexa Fluor 488 secondary antibody; DNA was counter-stained with DAPI. Pictures were collected at 20× magnification. **(B)** The number of cells positively stained with cleaved caspase-3 were counted in a population of at least 287 cells and the results are shown as the proportion of positive cells between treatment and control groups. The experiments were repeated 6 times. Statistical significance was calculated by a Chi-square/Fisher exact test (*=p≤0.05; **=p≤0.01). **(C)** Cells stably expressing pLKO.1, or shCDK4 were unirradiated (0), irradiated at 2 or 4 Gy. Protein lysates were prepared 48 hours later and were subjected to Western blot with anti-cleaved PARP antibody. β-actin was used as a loading control. **(D)** The CDK4/6 inhibitor PD0332991 was added to cells stably expressing pLKO.1 (100 nM, 500 nM and 1000 nM for MCF10A, MDA-MB-231 and MDA-MB-468, respectively) and unirradiated (0), irradiated at 2 or 4 Gy. Protein lysates were prepared 48 hours later and were subjected to Western blot with anti-cleaved PARP antibody. β-actin was used as a loading control. Levels were normalized based on β-actin, and are indicated as fold-induction relative to non-irradiated pLKO.1 controls.

Another mechanism that can explain the radiosensitization of cells after CDK4 silencing is that its loss potentially enhances frequencies of apoptosis. A pilot experiment established the window of maximal apoptosis following irradiation. Cell lines were either non-irradiated (basal), or received a single dose (2 Gy) of radiation and were collected at different times post-irradiation (0, 6, 12, 24 and 48 hours). Commitment to apoptosis was detected by immunocytochemistry against the apoptotic effector cleaved caspase-3 [75] (Figure 4A). This experiment revealed a gradual increase in the percentage of apoptotic cells, which became maximal at 48 hours (Additional file 3). Silencing of CDK4 caused elevation in apoptosis following irradiation in all cell lines tested, while knockdown of CDK2 did not lead to any changes in frequencies of apoptosis. Based on the results from the initial experiment, cells were irradiated with 0, 2, and 4 Gy, and analyzed 48 hours post-irradiation. Those experiments confirmed a significant elevation of apoptosis in cells silenced for CDK4 (Figure 4B). While MCF10A cells silenced for CDK4 displayed a significant proportion of caspase-3-positive cells under non-irradiated and with 2 Gy radiation, MDA-MB-468 cells silenced for CDK4 displayed a significant elevation in apoptosis only when irradiated at 2 and 4 Gy. Even though the proportion of apoptotic cells in MDA-MB-231 silenced for CDK4 were higher than controls in the basal and the 4 Gy groups, they did not reach statistical significance.

To establish whether the increases in apoptosis caused by radiation in cells silenced for CDK4 could be detected with an independent apoptosis marker, cells were subjected to Western blots using cleaved PARP (Figure 4C). In accordance with cleaved caspase-3 immunocytochemisty, higher levels of cleaved PARP were detected in MCF10A (6 to 11 fold changes) cells silenced for CDK4 compared to vector control. Consistent with the small increases in apoptosis with cleaved caspase-3, cleaved PARP levels were slightly elevated in MDA-MB-231 silenced for CDK4 relative to pLKO.1 controls. Likewise, we detected slightly elevated cleaved PARP levels in MDA-MB-468 shCDK4 cells irradiated with 2 Gy relative to MDA-MB-468 pLKO.1 cells. In contrast, no difference was found in cleaved PARP between MDA-MB-468 shCDK4 and MDA-MB-468 pLKO.1 cells upon irradiation with 4 Gy.

To establish whether radiosensitization also occurred through autophagy, we probed Western blots with an antibody against LC3A/3B. In MCF10A cells, only the lower band, which is the indicator of autophagy, was detected with no great difference among samples. Both inactive and active bands were observed in MDA-MB-231 and MDA-MB-468 cells, again with no great differences among samples (Additional file 4A). Finally, to investigate if the CDK4/6 kinase inhibitor, PD0332991, has a similar effect on inducing apoptosis or autophagy, MCF10A, MDA-MB-231 and MDA-MB-468 cells were treated at their respective IC50s [64] of 100 nM, 500 nM, and 1000 nM PD0332991, irradiated after 24 hours, and analyzed after 48 hours for cleaved PARP (Figure 4D). Unlike biological knockdown, levels of cleaved PARP did not increase in MCF10A pLKO.1 cells treated with PD0332991. Instead, high levels of cleaved PARP (2 to 3 fold) were detected in MDA-MB-468 pLKO.1 cells treated with PD0332991 compared to untreated cells. Again, LC3A/3B levels were not changed greatly among any of the cell lines, and did not change in the presence or absence of CDK4 (Additional file 4B). These results demonstrate that knockdown of CDK4 and chemical inhibition of CDK4/CDK6 lead to different outcomes, as the inhibitor is protective of apoptosis in irradiated MCF10A and MDA-MB-231 cells, while promoting apoptosis in MDA-MB-468 cells. Overall, the results also demonstrate that the primary mechanism of cell death in this system is through apoptosis and not autophagy.

### Knockdown of CDK4 results in reduced phosphorylation of BAD (ser136) and upregulation of protein phosphatase type 2A (PP2A)

**Figure 5:**
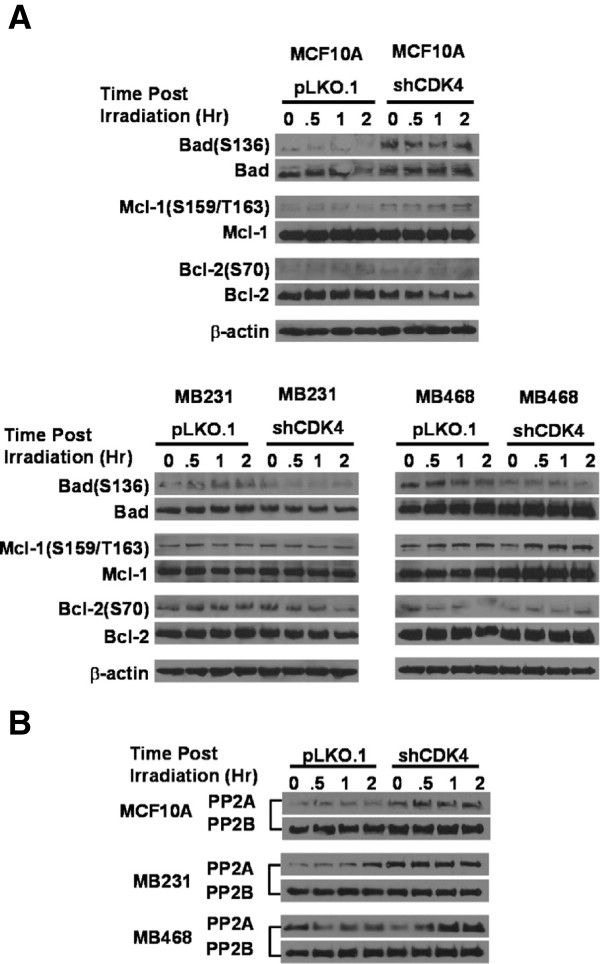
**Downregulation of Cdk4 results in impaired phosphorylation of BAD (ser136) and upregulation of protein phosphatase type 2A (PP2A).** Cells stably expressing pLKO.1 or shCDK4 were irradiated at 2 Gy. Protein lysates were then prepared at different times (0, 0.5, 1 & 2 hours) post-irradiation and were immunoblotted with anti-Bad(ser136), anti-Mcl-1(ser159/thr163) and anti-Bcl-2(ser70) antibodies, and their counterparts detecting total levels of those proteins. **(B)** The levels of PP2A and PP2B were further determined by immunoblotting with anti-PP2A and anti-PP2B antibodies. Cells transfected with pLKO.1 were used as control. The β-actin controls for **(B)** is the same as for **(A)**.

To assess why cells knocked down for CDK4 are prone to apoptosis upon irradiation, we performed a screen of various Bcl family members, with antibodies against the phosphorylated forms of those proteins, as well as total protein levels (Figure 5A). The lysates were obtained from cells pre-treated with Calyculin-A inhibitors, which inhibits phosphatase 1, 2A and 2B; the limitation of such method is that it increases the basal levels of most serine/threonine phosphorylation in cells. This method has been successfully used to detect phosphorylation of CDK2 or CDK4 targets, including NPM [73,76,77]. There were no significant changes in the levels of Bcl-2 (ser70) or Mcl-1 (ser159/thr163), or in the total levels of those proteins. However, lower levels of phospho-Bad (ser136) were observed in all irradiated ER-PR-HER2- cells expressing shCDK4, while MCF10A cells had elevated levels of phospho-Bad (ser136). Bad is a proapoptotic member of the Bcl-2 family that promotes cell death by displacing Bax from binding to Bcl-2 and Bcl-xL [78]. Phosphorylation of Bad at ser112 or ser136 via AKT inhibits the apoptotic activity of Bad by promoting binding of Bad to 14-3-3 proteins to prevent an association between Bad with Bcl-2 and Bcl-xl [79]. The dephosphorylation of Bad is mainly accomplished by various protein phosphatases [80-82]. We speculated that Bad ser136 was being dephosphorylated through the increased activity and/or levels of a phosphatase. It has been reported that phospho-ser136 is more efficiently hydrolyzed by PP1? and PP2A relative to PP2B and PP2C [82]. To find out what is responsible for the dephosphorylation of Bad at ser136, we detected the relative protein levels of the protein phosphatases PP1α, PP2A, and PP2B. As shown in Figure 5B, the basal levels of PP2A at time 0 were immediately upregulated in MCF10A and MDA-MB-231 cells lacking CDK4 upon irradiation, and the levels remained upregulated. As for MDA-MB-468 cells, the levels of PP2A increased gradually following irradiation. These experiments suggested that downregulation of CDK4 lead to increases in PP2A, which dephosphorylates phospho-Bad in ser136.

### Silencing the catalytic subunit of PP2A does not lead to increased PARP cleavage in irradiated cells silenced for CDK4

**Figure 6:**
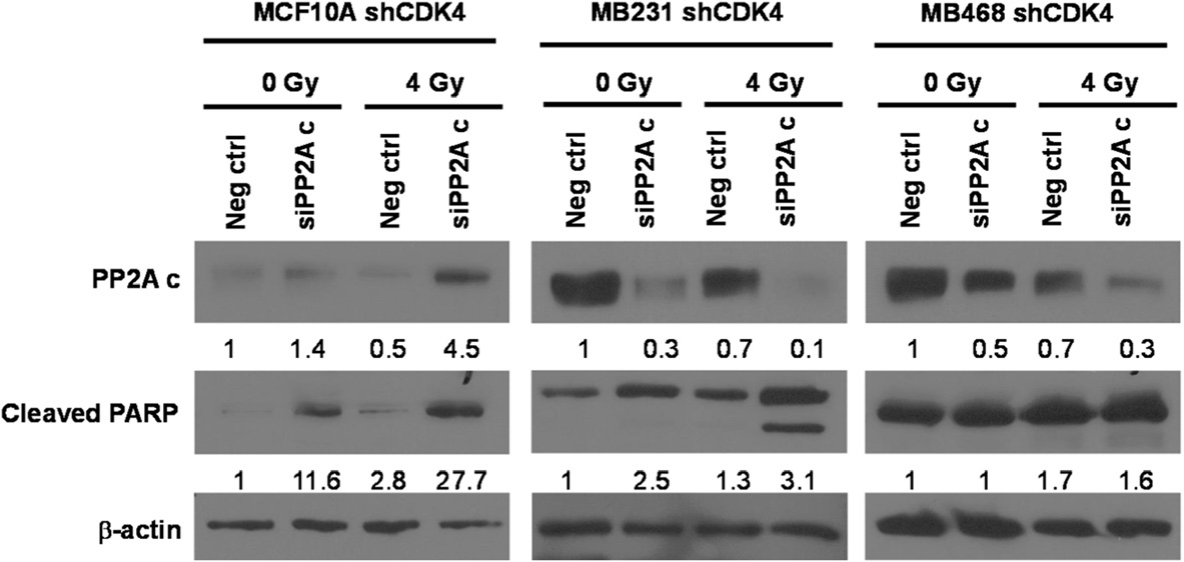
**Silencing PP2A does not change cleaved PARP level in Cdk4 silencing cells.** A cocktail of siRNAs targeting the PP2A catalytic unit was transiently transfected into cells stably expressing shCDK4 and cells were irradiated at 0, 2 and 4 Gy after 48 hours transfection. Protein lysates were then prepared at 48 hours post-irradiation and were immunoblotted with PP2A, cleaved PARP. β-actin was used as a loading control. Protein levels were normalized based on β-actin, and are indicated as fold-induction relative to scrambled siRNA controls.

To investigate whether silencing of PP2A reverses apoptosis in irradiated cells silenced for CDK4, the PP2A catalytic unit was knocked down using siRNAs (Figure 6). PP2A knock down was efficient in MDA-MB-231 (70%) and MDA-MB-468 cells (50%) and irradiation further decreased PP2A level in both negative control (30% less compared to nonirradiated) and cells silenced for PP2Ac (40-60% less compared to nonirradiated control). Overall, silencing PP2A did not change upregulated cleaved PARP levels in MDA-MB-468 shCDK4. On the other hand, it elevated cleaved PARP in MDA-MB-231 shCDK4 cells level compared to the negative controls. Knockdown PP2A in MCF10A cells silenced for CDK4 under the same conditions was not successful. The autophagy marker LC3A/3B levels were not changed greatly among cell lines and samples (Additional file 4C). This data indicates that downregulation of PP2A does not reverse apoptosis in cells knocked down for CDK4. We conclude that apoptosis in irradiated cells silenced for CDK4 does not involve the PP2A catalytic subunit.

## Discussion

The G1 phase CDKs, (CDK2, CDK4, and CDK6) display specific functions in terms of development and redundant functions in terms of cell cycle regulation [23,24,83]. The redundant nature of the G1 phase CDKs is best illustrated by work showing that cells can proliferate in the absence of CDK2 and CDK4, and that CDK1 activity is required for cell cycle progression [84,85]. However, injured hepatocytes do not require CDK1 to proliferate [86]. Another important function of the CDKs is in DNA repair. Specifically, cyclin D1, CDK4 [58,59,87], cyclin A1, and CDK2 have been implicated in DNA repair [88-92]. In fact, the activities of all CDKs are required for efficient DNA repair [93]. Even though CDK2 and CDK4 are largely dispensable in the entry and progression through S phase, they are important mediators of mammary tumorigenesis in mice, as ablation of either CDK2 or CDK4 can prevent mouse mammary tumorigenesis triggered by HER2/Neu [94,95]. Also, CDK2 and CDK4 can differentially mediate centrosome amplification depending on the oncogenic or cellular context [96]. While cyclin D1/CDK4 specifically mediates centrosome amplification triggered by H-Ras^G12D^, H-Ras^G12D^&c-Myc [97], and in HER2+ breast cancer cells [76], silencing of cyclin E or CDK2 have no impact on centrosome amplification in the HER2/Ras systems. In contrast, both CDK2 and CDK4 mediate centrosome amplification and chromosome instability in p53 deficient cells [73].

Overall, the ability of CDK2 and CDK4 to mediate various malignant phenotypes that play important roles in cancer biogenesis has prompted the development of CDK2 or CDK4 specific inhibitors. Those inhibitors have proven to be effective in a variety of breast cancer cell lines. For example, ER+ luminal cells that overexpress cyclin D1 and Rb and display downregulated p16 respond well to cell cycle inhibition by the CDK4/CDK6 specific inhibitor PD-0332991, while non-luminal/basal cells are refractive to growth inhibition [64]. It has been reported that the Rb status dictates the early responses to that inhibitor [98]. Even though the CDK4/CDK6 inhibitor has shown promise in preclinical trials, cells acquire resistance through the upregulation of CDK2 activity [98]. A major limitation of PD-0332991 is that while it is effective in extinguishing mammary tumors, its ability to block the cell cycle interferes with chemotherapy [99,100].

In the present study, we addressed whether the G1/S cell cycle regulatory machinery influences radioresistance in MCF10A, ER-PR-HER2-, and ER-PR-HER2+ cells. There is a precedent for the role of ectopic CDK2 and CDK4 activities in imparting resistance or sensitivity to radiotherapy. For example, knockdown of cyclin E synergizes with doxorubicin to enhance radioresistance in breast cancer cells lines [101]. Induction of cyclin A has previously been observed in cells treated with UV or irradiation [91,102]. Cyclin A null cells are radiosensitive and display impaired double strand break repair; cyclin A/CDK2 is involved in DNA repair following irradiation by phosphorylating KU-70 [91].

Various mechanisms have been postulated to explain how cyclin D1/CDK4 leads to radioresistance or radiosensitivity of various cells. Inducible expression of cyclin D1 in MCF7 breast cancer cells leads to radiosensitivity through activation of the p53 pathway [60]. Low dose irradiation promotes the free, cytoplasmic cyclin D1 accumulation in human keratinocytes, correlating with radioresistance; in this context, low level radiation disrupted the interaction of cyclin D1 with 14-3-3ζ [55]. Cytoplasmic cyclin D1 then interacts with Bax, suppressing the ability of Bax to induce apoptosis. In another example, long term fractionated irradiation of the human cancer cell lines HepG2 and HeLa induced radioresistance [59]. This radioresistance correlated with upregulation of cyclin D1 due to the stabilization of cyclin D1 by prevention of its proteolysis, achieved by the DNA PK/AKT/GSK3β pathway. In addition, they first proposed that ectopic expression of cyclin D1 leads to increased radioresistance in breast cancer cells by enhancing DNA repair in the radioresistant cells, as downregulation of cyclin D1 in this system abrogated enhanced DNA repair, resulting in radiosensitivity. This group showed using chemical inhibitors that cyclin D1 dependent radioresistance is reversed by AKT or CDK4 chemical inhibitors. A recent report also demonstrated that enhanced cyclin D1 results in enhanced DNA repair and radioresistance [58]. This report postulates that cyclin D1 promotes DNA repair by interacting with RAD51 within DNA repair foci; this interaction is promoted by radiation.

Our studies demonstrate that knockdown of CDK4 acts as a potent radiosensitizer, independently of the breast cancer molecular subtype. In our studies, we tested whether radiosensitization was a consequence of cell cycle inhibition by silenced CDK4, altered DNA break repair, or through the activation of an apoptotic program. We used various assays to show that knockdown of CDK4 did not influence the cell cycle of non-irradiated or irradiated cells. This is consistent with our previous observations that in MCF10A cells expressing control pLKO.1, H-Ras^G12V^, H-Ras^G12V^&c-Myc, or in HER2+ breast cancer cells, knockdown of CDK4 does not greatly influence DNA replication or cell cycle progression [76,97]. This is also consistent with our data showing that ablation of CDK4 does not alter the cell cycle of p53−/− mouse embryonic fibroblasts [73]. In contrast, co-inhibition of CDK4 and CDK6 using a chemical inhibitor interferes with the cell cycle, leading to protection against chemotherapy [99,100]. Our studies conducted with the PD0332991 CDK4/CDK6 inhibitor revealed a similar story, as treatment of MCF10A or MDA-MB-231 protected against radiation-induced apoptosis. This is contrary to our studies revealing that knockdown of CDK4 synergizes with radiation to induce apoptosis in breast cancer cells and MCF10A controls. The only cell in which radiation synergized with inhibition of CDK4/CDK6 was MDA-MB-468, a cell that is insensitive to growth inhibition by PD0332991. However, we do not think that insensitivity to growth inhibition is the sole cause of the sensitivity to apoptosis triggered by PD0332991 treatment, as we treated all cell lines exactly at IC50 to prevent complete growth inhibition. One potential explanation for the differences between knockdown of CDK4 and inhibition with PD0332991 is that while the chemical inhibits CDK4/CDK6 activity, the shCDK4 may disrupt the protein:protein interactions of CDK4 with cyclin D, p21, p27, p15, p16, p18 and p19, leading to modulation of the activities of other CDKs.

In contrast to studies showing that increased cyclin D1 activity results in radioresistance through increasing cellular DNA repair capacity, all breast cancer cells tested in this study efficiently repaired DNA breaks irrespective of CDK4 levels, as indicated by the efficient clearance of H2AX foci after radiation. A recent study is consistent with our results, as chemical inhibition of CDK4 and CDK6 in breast cancer cells did not influence double-strand break repair after irradiation [99]. We speculate that the differences between cyclin D1 and CDK4/CDK6 inhibition in relation to DNA repair is because cyclin D1 displays functions that are independent of CDK4. For example, it can bind the promoters of genes involved in chromosome instability [103].

The presence of increased levels of cleaved caspase-3 and of cleaved PARP in all irradiated cells down regulated for CDK4 indicated that apoptosis was a significant driver of radioresistance in mammary epithelial and breast cancer cells. Although apoptosis is high in unirradiated MCF10A cells silenced for CDK4, levels of apoptosis rise significantly in those and in the breast cancer cells silenced for CDK4 upon irradiation. This suggests synergy between downregulation of CDK4 and irradiation in regards to apoptosis. To establish how absence of CDK4 cooperates with radiation to enhance cell death, we screened various Bcl family members. No changes in the levels of phosphorylation of the antiapoptotic members Bcl2 or Mcl1 were observed in the cells. In contrast, levels of phosphorylated Bad ser136 were sharply diminished Even though dephosphorylation of Bad in serine 136 is associated with apoptosis, we observe increased phosphorylation in irradiated shCDK4 MCF10A cells. The regulation of apoptosis is complex, involving various protein/protein interactions, as well as the post-translational modification of numerous proteins. Further studies must be performed in order to establish why MCF10A cells silenced for CDK4 display phosphorylated Bad Ser 136, and still apoptose. Bad ser136 phosphorylation is reversed by various phosphatases, including PP2A and PP1α [81,82]. In fact, silencing of CDK4 resulted in higher levels of PP2A, suggesting that it specifically dephosphorylates Bad ser136 upon irradiation PP2A is a protein complex with many cellular functions, including the regulation of apoptotic and mitogenic pathways [104-106], as well as the modulation of DNA repair [107]. Inhibition of PP2A by DNA tumor viruses is tightly linked to cellular transformation [108]. Also, the dephosphorylation of Bcl2 by PP2A promotes its anti-apoptotic activity; this event may enhance Bcl2’s oncogenic potential [109]. In wide contrast, PP2A’s proapoptotic activities have been mapped to its ability to dephosphorylate Bad [81,82] and Bax [110]. We did not observe significant dephosphorylation of Bcl2 in any of the cell lines, suggesting that upregulation of PP2A in breast cancer cells exclusively dephosphorylates Bad ser136 without significantly affecting other Bcl family members. Thus, our observation that downregulation of CDK4 resulted in decreased phosphorylation of Bad ser136 suggests that this event is primarily responsible for apoptosis and radioresistance. Additional experiments are required to address how CDK4 regulates PP2A levels; that regulation may result from increased protein stability, increased degradation, or enhanced de-repression of E2F target genes upon CDK4 silencing. Another important future area of exploration is whether and how the apoptotic program triggered by downregulation of CDK4 in conjunction with radiation is rooted in Rb/E2F dependent transcription.

Unfortunately, we were unable to show that ectopic expression of PP2Ac radiosensitizes breast cancer cells, or that its inhibition modulates radiation induced apoptosis in breast cancer cells. We conclude that apoptosis in cells knocked down for CDK4 is independent of PP2A. Further experiments are required to establish pathways signaling apoptosis in irradiated breast cancer cells knocked down for CDK4.

## Conclusion

We conclude that stable knockdown of CDK4 enhances the effects of radiation by enhancing apoptotic pathways. This result has implications for future therapies, as patients can be treated with radiation or chemotherapeutic agents and with doses of CDK4/CDK6 inhibitors that do not block the cell cycle. In that scenario, suppression of CDK4/CDK6 activity would allow cell cycle progression, while synergizing with radiation to increase cell death.

## Materials and methods

### Cell culture

A human mammary epithelial cell line (MCF10A) and breast cancer cell lines (ER-PR-HER2-:HCC1806, HS578, MDA-MB-231, MDA-MB-468; ER-PR-HER2+: SKBR3 and HCC1954), were purchased from ATCC (Rockville, MD), or obtained through collaborators. The molecular markers and various other characteristics of the breast cancer cells used in this study have been previously described [111]. HCC1806, HCC1954, and SKBR3 cells were cultured in RPMI supplemented with 10% fetal bovine serum (FBS). HS578, MDA-MB-231 and MDA-MB-468 cells were cultured in DMEM supplemented with 10% FBS. MCF10A cells were cultured in DMEM/F12 supplemented 10% FBS, 20 ng/ml EGF, 100 ng/ml cholera toxin, 0.01 mg/ml insulin, and 500 ng/ml hydrocortisone. All cell lines were cultured at 37°C in a humidified atmosphere (5% CO_2_).

### Colony forming assay

Two thousand five hundred cells were plated per well in a 6-well culture plate or one thousand cells were plated per well in a 12-well cell culture plate (Day 0) in triplicates. After 24 hours (Day 1), cells were irradiated at a dose of 0, 1, 2, 4, 6, or 8Gy in an X-RAD 320 irradiator (Precision X-Ray, CA). After an average of 8 days, cells were fixed with 75% ethanol and stained with 1% crystal violet, and colonies containing more than 50 cells (established by microscopy) were counted manually. Plating efficiency was calculated by dividing the number of colonies in the unirradiated group divided by the number of colonies plated and multiplying by 100.

### Lentiviral shRNA transduction

The shRNA constructs against CDK2 or CDK4 were cloned into the lentiviral vector pLKO.1, which are commercially available from OPEN Biosystems. Lentiviral infections were done as described in our publications [97]. Briefly, cells were plated in regular medium at 50% confluence on day 1. This was followed by changing into medium containing 5 μg/ml polybrene and infection of cells by adding undiluted shRNA lentiviral particles to the culture on day 2. Stable clones expressing the shRNA were obtained by splitting cells 1:5 and culturing in medium containing puromycin.

### Immunocytochemistry assays

Immunofluorescence was performed by following our published protocols [73,97]. Cells were plated at 4×10^4^ per well into two-well tissue culture chamber slides and received 2 Gy irradiation after cells attached. Cells were then fixed in cold 4% paraformaldehyde at different times postirradiation (from 0 to 48 hr), washed in PBS, permeabilized in a 1% NP-40–PBS solution, and blocked in 10% goat serum. Commitment to apoptosis was detected by using an antibody against cleaved caspase-3 (9661, Cell Signaling). DNA breaks were detected using an antibody against phosphorylated histone 2A variant X (γ-H2AX) (ab2893, Abcam). Cell proliferation was examined using an antibody against Ki-67 (ab15580, Abcam). The BrdU assay was used to detect cells in S phase and was done by pulsing cells with 20 μM BrdU (51-7581KZ; BD Pharmingen) 30 minutes before harvest and detecting with an anti-BrdU antibody (NA61; Calbiochem). To detect mitosis, cells were plated at 1×104 per well into four-well tissue culture chamber slides and received 0, 2 and 4 Gy irradiation 24 hours after plating and incubated 48 hours and phosphor-histone 3 (Ser 28, 9713, Cell Signaling) antibody was used to detect mitosis. Secondary antibodies are conjugated with Alexa Fluor 555 or Alexa Fluor 488. Cells were counterstained with DAPI. Frequencies of the various phenotypes are done by counting the number of antigen-positive cells divided by the total number of cells; unless otherwise indicated, we count at least 200 cells per group. For mitosis, over 300 cells per group were counted.

### Western blots

Western blotting was performed according to published protocols [73,97]. Protein lysates were obtained by incubating cells in lysis buffer (50 mM HEPES, 250 mM KCl, 0.1 mM EDTA, 0.1 mM EGTA, 0.1% NP-40, 10% glycerol) containing protease inhibitors (04693124001, Roche) for 30 min at 4°C. Calyculin phosphatase inhibitor (Millipore 19-139v) was added to the culture medium at a concentration of 100 nM, 30 minutes before harvest for the detection of phosphorylated proteins. Samples were denatured at 95°C for 5 min in sodium dodecyl sulfate (SDS) sample buffer, resolved by SDS polyacrylamide gel electrophoresis and transferred to polyvinylidene difluoride membranes (Bio-Rad). The blots were incubated in blocking buffer (5% nonfat dry milk in 1X Tris-buffered saline plus 0.1% Tween 20 [TBS-T]) for 1hr and were then probed overnight at 4°C with the primary antibodies. The blots were then rinsed in 1X TBS-T and incubated with the appropriate horseradish peroxidase conjugated secondary antibodies at room temperature. The blots were then rinsed in 1X TBS-T and the antibody-antigen complex was visualized using Lumigen TMA-6 (RPN2135v1 and RPN2135v2; GE Healthcare).

### Antibodies and inhibitors

The antibodies used in the various Western blotting experiments were as follows: CDK2 (sc-163, Santa Cruz), CDK4 (2906, Cell Signaling), p21^Waf1^ (sc-397, Santa Cruz), p27^Kip1^ (sc-528, Santa Cruz), β-actin (4970, Cell Signaling), cyclin A (ab38, Abcam), cyclin D1 (2922, Cell Signaling), cyclin E (sc-481, Santa Cruz), Phospho-Bcl-2 ser70 (2827, Cell Signaling), Bcl-2 (OP60, Calbiochem), Phospho-Mcl ser159/thr163 (4579, Cell Signaling), Mcl-1 (sc-819, Santa Cruz), Phospho-Bad ser136 (06–846, Upstate), Bad (1541–1, Epitomic), Phospho-AKT Ser 473 (Cell Signaling), PP1α (2582, Cell Signaling), PP2A (2259, Cell Signaling) and PP2B (2614, Cell Signaling), cleaved PARP (Asp214, 5625, Cell Signaling). The CDK4/6 inhibitor PD0332991 (Axon1505) was purchased from Axon Medchem. When indicated, ImageJ software was used to quantify protein levels of Western blots (NIH).

### Flow cytometry

Cells were irradiated at 2 Gy, pulsed with BrdU (added at a final concentration of 10 μM 30 min prior to harvest) and harvested at various time points (6, 12, and 24 hours). Timepoints were chosen based on our preliminary and supplementary data. A unirradiated control was also collected. 1×10^6^ cells were transferred to 5ml round bottom tubes (352235, BD), and cell staining was performed according to the protocol included with the “Apoptosis, DNA damage and Cell Proliferation kit” (562253, BD) or stained with 7-AAD (559925, BD Biosciences). BrdU was detected using PerCP-Cy™5.5 Mouse Anti-BrdU (51–9007682, BD), PARP cleavage was identified with PE Mouse Anti-Cleaved PARP (Asp214) Antibody (51–9007684, BD) and γH2AX was distinguished using Alexa Fluor® 647 Mouse Anti-H2AX (pS139) (51–9007683, BD). A LSRII flow cytometer (BD) was used to read each sample. Compensation was performed using Anti-Mouse Ig, κ/Negative Control (FBS) Compensation Particles Set (BD, 552843) and samples were analyzed using Flowjo v7.6.5 and v10.4 (Tree Star).

### Statistical methods

For the colony forming assay, a dose response analysis was conducted, in which the dose response relationship was assumed to be the commonly used four parameter logistic function [112]. The data was normalized relative to the baseline dose level=0 Gy (no radiation), and the percentage of control was used as the primary outcome in the analysis. IC50, the radiation dose that is required for 50% reduction from baseline dose level, was the parameter of interest and compared accordingly. The higher value of IC50, the higher the resistance. A proper data transformation, Box- Cox transformation, [113] was applied to assure the fitted model meeting the statistical assumptions. All data management and statistical analyses were done in R [114]. T-tests were done in Excel. The statistical significance level of all tests was set at 0.05.

For the flow cytometry assays (DNA content and BrdU) the data was aligned by the six-hour gates. The two measurements (percentages of positive cells) per each run are averaged at each time point and the averaged measurement at each of 6 hours, 12 hours and 24 hours after radiation is divided by the averaged measurement at baseline. The mean percentage of the positive cells relative to no radiation is used for analyses, named fold-change to no radiation. The main goal of this data analysis is to compare the fold change between control and treatment groups at each time point, within each cell line, biomarker, and gate. The fold change is compared between control group and treatment group (pLKO.1 vs. shCDK4) at each time point within each cell line, biomarker, and gate using ANOVA (Analysis of Variance) to estimate the mean and standard error for the fold change to non-radiation. For the cleaved caspase-3 assay, observations (number of positive or negative cells) are combined within each radiation dose of 2 Gy, 4 Gy or no radiation treated cells for each control group and treatment group and then the Chi-square test or Fisher’s exact test is used to compare the proportions of positive and negative cells between treatment and control groups. Analysis unit is a cell. The significance levels are set at 0.05 for all tests. The SAS statistical package V9.3 (SAS Institute, Inc., Cary, North Carolina) is used for data managements and analyses.

### Ethical approval

No research on humans or animals were conducted. All human cell lines were purchased from the publically available American Type Culture Collection (ATCC), or obtained through collaborators.

## Abbreviations

CDK: Cyclin-dependent kinase; Gy: Gray.

## Competing interests

The authors declare they have no competing interests.

## Authors’ contributions

KRH: Performed the radiation and flow cytometry experiments to detect DNA proliferation, apoptosis and DNA breaks. Performed caspase immunoflourecent staining assays. Was involved in finalizing the writing of the manuscript. XZ: Performed western blots and caspase, BrdU, Ki67 and γ-H2AX preliminary and supplementary experiments in the manuscript. Participated in early drafts of the manuscripts. MYL Performed Western blots for cleaved PARP and LC3A/3B in the presence of shCDK4 and the CDK4/CDK6 inhibitor, immunocytochemistry for phospho-histone 3 Contributed and modified figures and was involved in manuscript editing. STK: Performed the analysis showing that breast cancer cell lines are resistant to radiation. Showed that silencing CDK4 radiosensitized breast cancer cells. MKHP: Developed cell lines stably silenced for CDK4 and performed Western blots showing downregulation of CDK4. Edited the manuscript. SSZ: Developed the irradiation protocols for the radiation sensitivity and colony formation experiments. YL: Biostatistician that performed many of the statistical assays in the manuscript. RMO: Designed some of the experiments and provided various cell lines. XD: Provided antibodies and designed the experiments that lead to the Western blot analyses of various apoptotic proteins. HIS: Designed the experiments in the manuscript, edited the manuscript, and established all collaborations in the manuscript. All authors read and approved the final manuscript.

## Supplementary Material

Additional file 1 **Radiosensitivity imparted by downregulated Cdk4 is not caused by impaired proliferation. (A)** Cells stably expressing control pLKO.1, shCDK2 or shCDK4 were unirradiated (Basal) or irradiated at 2 Gy. Cells were pulse-labeled with BrdU following different times post-irradiation (0, 6, 12, 24, & 48 hours), and were subjected to immunostaining with an anti-BrdU antibody, and an Alexa Fluor secondary antibody. Nuclei were counter-stained with DAPI. The number of cells positively stained with BrdU was counted in 200 cells per group, and the results are shown as the average±standard deviation from two independent experiments. **(B)** Cells stably expressing pLKO.1, shCDK2 or shCDK4 were unirradiated (Basal), or irradiated at 2 Gy. Cells collected from different time points (0, 6, 12, 24, & 48 hours) post-irradiation were subjected to immunostaining with anti-Ki67 antibody, an Alexa Fluor secondary and the DNA was counter-stained with DAPI. The number of cells positively stained with Ki67 was counted in 200 cells per group, and the results are shown as the average±standard deviation from two experiments. Click here for file

Additional file 2 **Downregulation of Cdk4 does not alter rates of DNA break repair.** Non-infected cells and cells stably expressing pLKO.1, shCDK2 or shCDK4 were irradiated at 2 Gy. The basal levels were determined in unirradiated cells. Cells were fixed at different times (0, 6, 12, 24, & 48 hours) post-irradiation and were subjected to immunostaining with anti-H2A.X antibody (γ-H2AX) and an Alexa Fluor 555 secondary antibody; DNA was counter-stained with DAPI **(A)**. Blue cells represent nuclei, while the red cells (arrows) represent cells expressing γ-H2AX. Pictures were taken at a 65× magnification. **(B)** The number of cells positively stained with γ-H2AX was counted in 200 cells per group, and the results are shown as the average±standard deviation from two experiments. Click here for file

Additional file 3 **Silencing of Cdk4 promotes apoptosis.** Cells stably expressing pLKO.1, shCDK2 or shCDK4 were irradiated at 2 Gy. The basal levels were determined in unirradiated cells. Cells were fixed at different times (0, 6, 12, 24, & 48 hours) post-irradiation and were subjected to immunostaining with anti-cleaved caspase-3 antibody and an Alexa Fluor 555 secondary antibody; DNA was counter-stained with DAPI. The number of cells positively stained with cleaved caspase-3 was counted in 200 cells per group, and the results are shown as the average±standard deviation from two experiments. Click here for file

Additional file 4 **CDK4 silencing did not change degree of autophagy. (A)** Cells stably expressing control pLKO.1 and shCDK4 were irradiated at 0, 2 and 4 Gy. Protein lysates were prepared after 48 hours post irradiation and were subjected to Western blot with an anti-LC3A/3B antibody. β-actin was used as a loading control. **(B)** Cells stably expressing control pLKO.1 were treated with the CDK4/6 inhibitor PD0332991 and irradiated at 0, 2 and 4 Gy. Protein lysates were prepared after 48 hours post irradiation and were subjected to Western blot with an anti-LC3A/3B antibody. β-actin was used as a loading control. **(C)** Cells stably expressing shCDK4 was transfected with siRNA targeting the PP2A catalytic unit for 48 hours and irradiated at 0, 2 and 4 Gy. Protein lysates were prepared after 48 hours post irradiation and were subjected to Western blot with an anti-LC3A/3B antibody. β-actin was used as a loading control. Click here for file
