# Supplementary figures and images for: Silencing CDK4 radiosensitizes breast cancer cells by promoting apoptosis
Source: Cell Div. 2013 Jul 25;8:10. doi: 10.1186/1747-1028-8-10 (PMC3733890; doi:10.1186/1747-1028-8-10)

**A**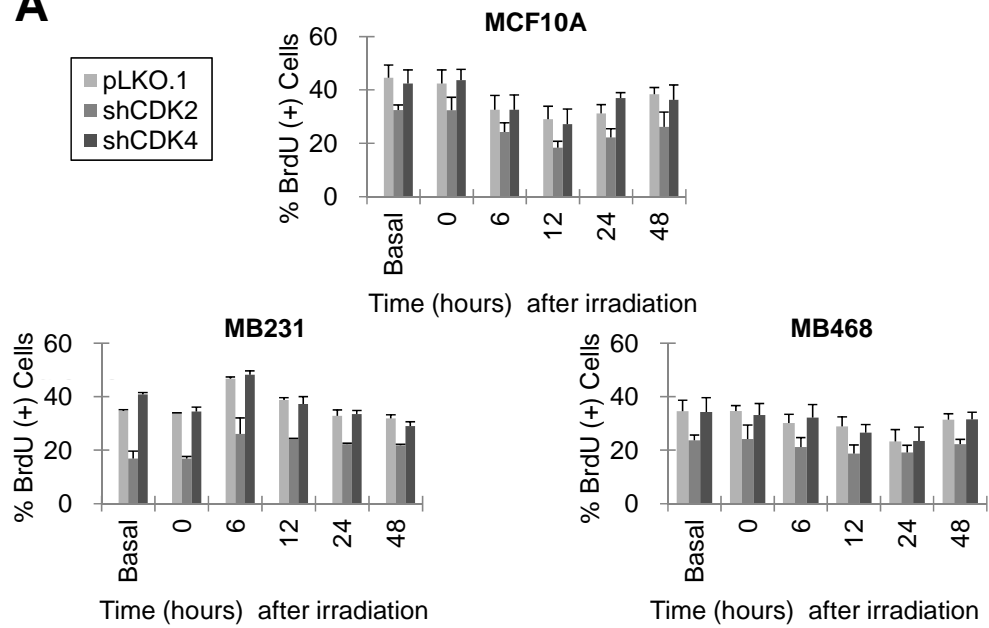**B**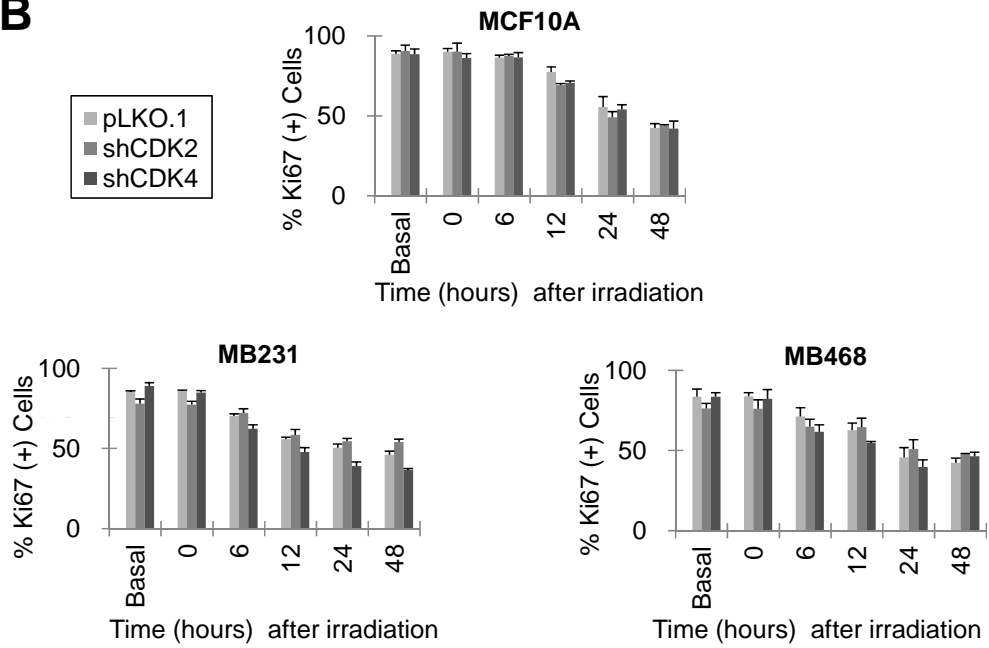

Supplement: Additional file 1 — Radiosensitivity imparted by downregulated Cdk4 is not caused by impaired proliferation. (A) Cells stably expressing control pLKO.1, shCDK2 or shCDK4 were unirradiated (Basal) or irradiated at 2 Gy. Cells were pulse-labeled with BrdU following different times post-irradiation (0, 6, 12, 24, & 48 hours), and were subjected to immunostaining with an anti-BrdU antibody, and an Alexa Fluor secondary antibody. Nuclei were counter-stained with DAPI. The number of cells positively stained with BrdU was counted in 200 cells per group, and the results are shown as the average±standard deviation from two independent experiments. (B) Cells stably expressing pLKO.1, shCDK2 or shCDK4 were unirradiated (Basal), or irradiated at 2 Gy. Cells collected from different time points (0, 6, 12, 24, & 48 hours) post-irradiation were subjected to immunostaining with anti-Ki67 antibody, an Alexa Fluor secondary and the DNA was counter-stained with DAPI. The number of cells positively stained with Ki67 was counted in 200 cells per group, and the results are shown as the average±standard deviation from two experiments. [file 1747-1028-8-10-S1.pdf]

**A**

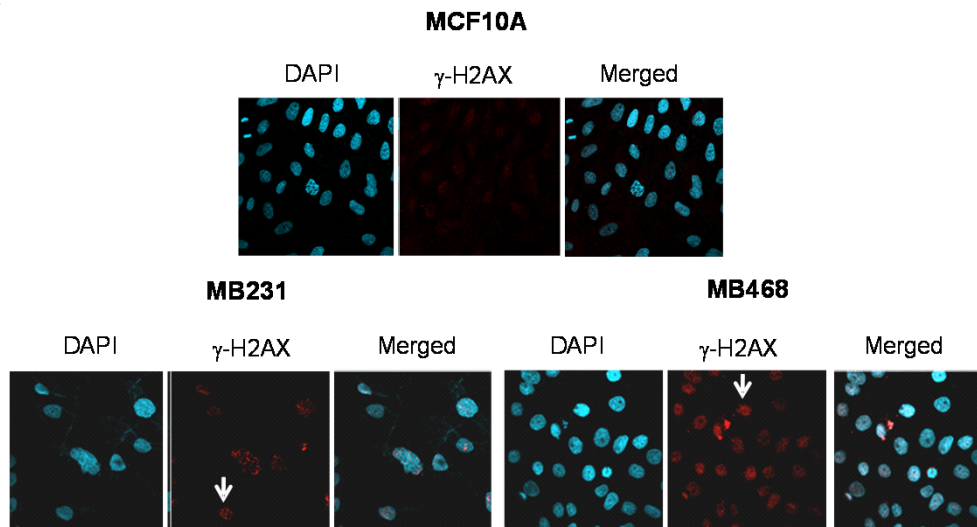

**B**

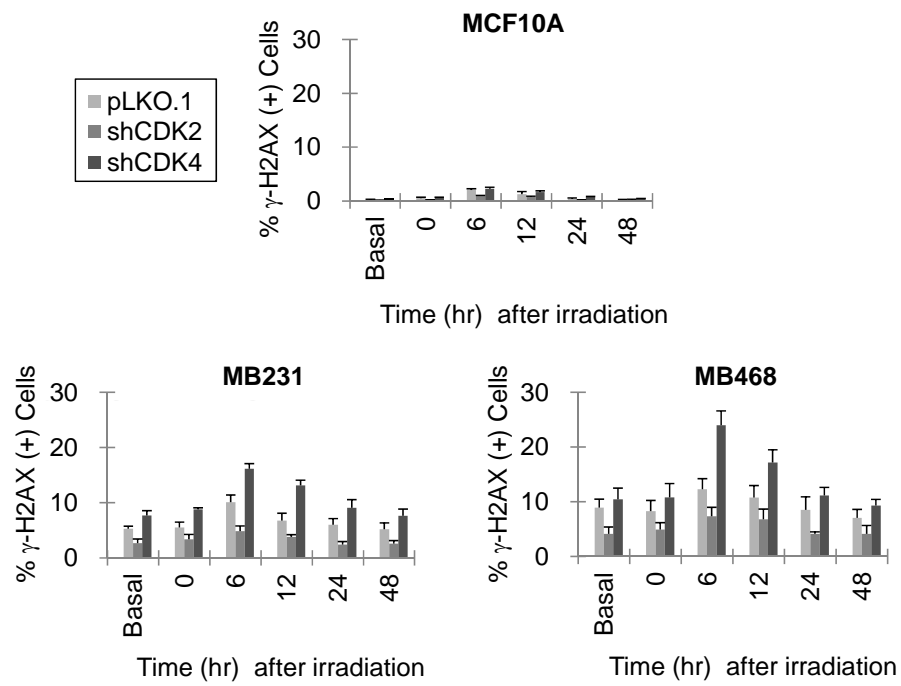

Supplement: Additional file 2 — Downregulation of Cdk4 does not alter rates of DNA break repair. Non-infected cells and cells stably expressing pLKO.1, shCDK2 or shCDK4 were irradiated at 2 Gy. The basal levels were determined in unirradiated cells. Cells were fixed at different times (0, 6, 12, 24, & 48 hours) post-irradiation and were subjected to immunostaining with anti-H2A.X antibody (γ-H2AX) and an Alexa Fluor 555 secondary antibody; DNA was counter-stained with DAPI (A). Blue cells represent nuclei, while the red cells (arrows) represent cells expressing γ-H2AX. Pictures were taken at a 65× magnification. (B) The number of cells positively stained with γ-H2AX was counted in 200 cells per group, and the results are shown as the average±standard deviation from two experiments. [file 1747-1028-8-10-S2.pdf]

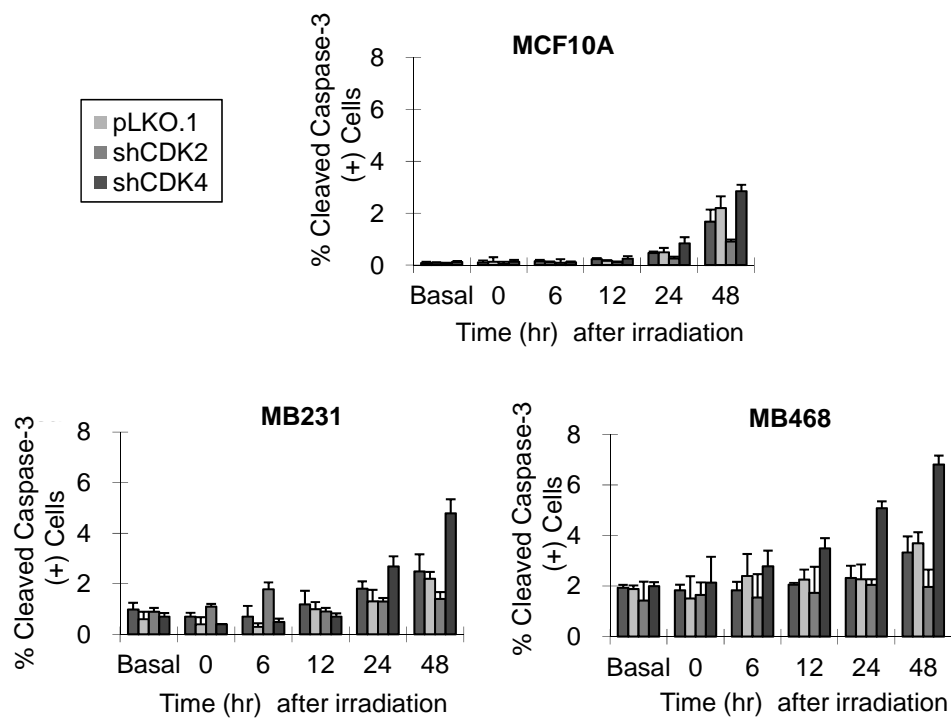

Supplement: Additional file 3 — Silencing of Cdk4 promotes apoptosis. Cells stably expressing pLKO.1, shCDK2 or shCDK4 were irradiated at 2 Gy. The basal levels were determined in unirradiated cells. Cells were fixed at different times (0, 6, 12, 24, & 48 hours) post-irradiation and were subjected to immunostaining with anti-cleaved caspase-3 antibody and an Alexa Fluor 555 secondary antibody; DNA was counter-stained with DAPI. The number of cells positively stained with cleaved caspase-3 was counted in 200 cells per group, and the results are shown as the average±standard deviation from two experiments. [file 1747-1028-8-10-S3.pdf]

**A**

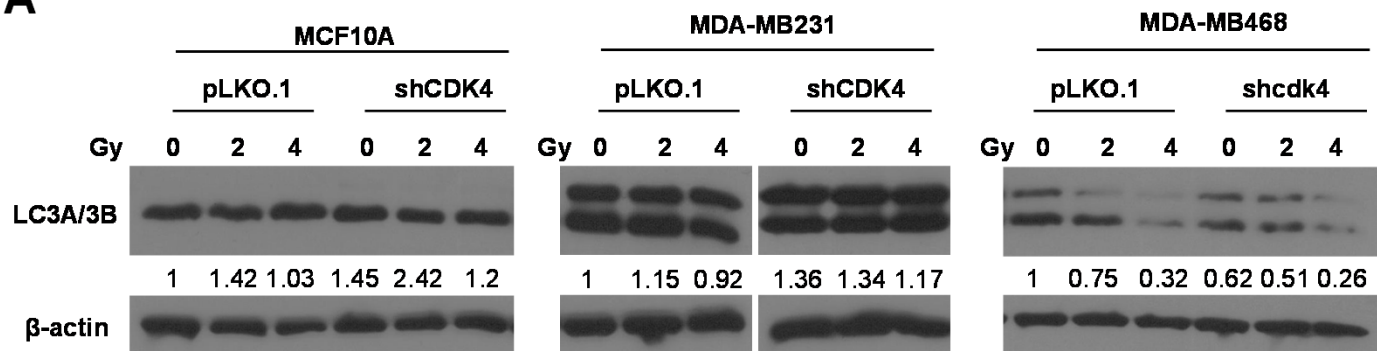

**B**

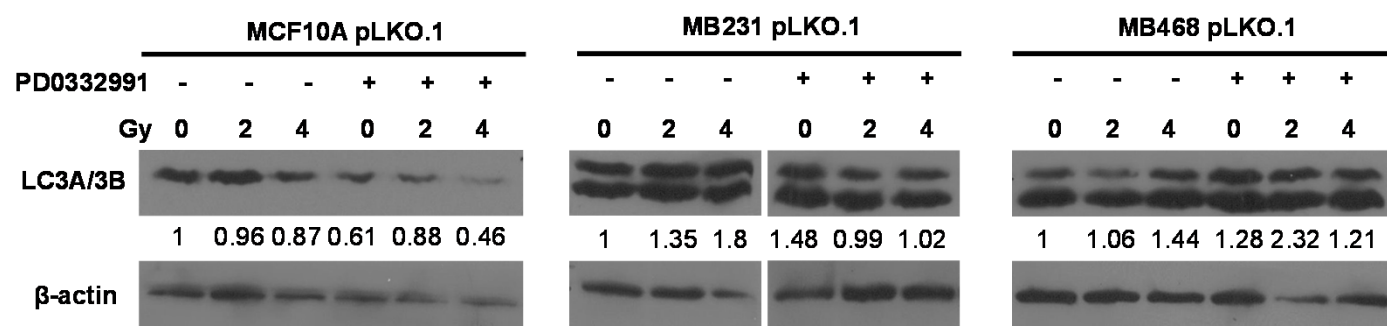

**C**

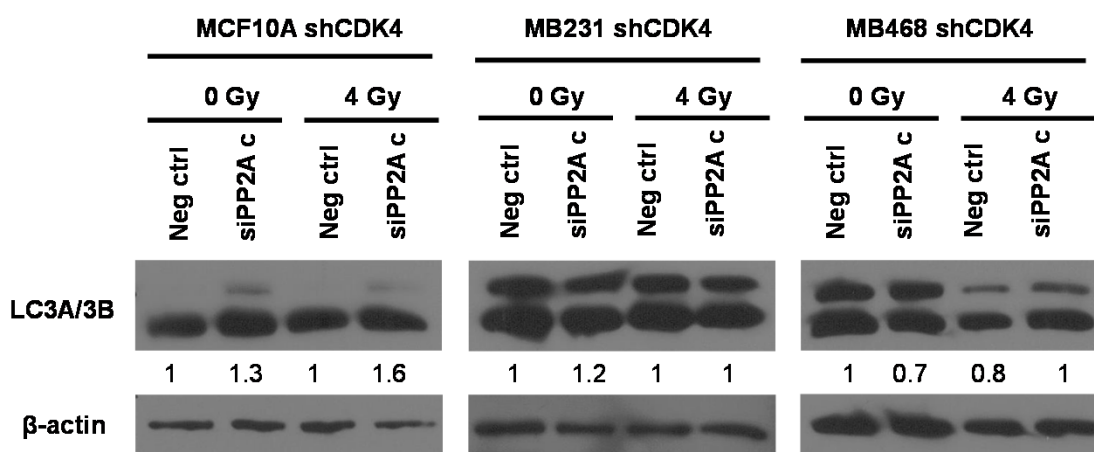

Supplement: Additional file 4 — CDK4 silencing did not change degree of autophagy. (A) Cells stably expressing control pLKO.1 and shCDK4 were irradiated at 0, 2 and 4 Gy. Protein lysates were prepared after 48 hours post irradiation and were subjected to Western blot with an anti-LC3A/3B antibody. β-actin was used as a loading control. (B) Cells stably expressing control pLKO.1 were treated with the CDK4/6 inhibitor PD0332991 and irradiated at 0, 2 and 4 Gy. Protein lysates were prepared after 48 hours post irradiation and were subjected to Western blot with an anti-LC3A/3B antibody. β-actin was used as a loading control. (C) Cells stably expressing shCDK4 was transfected with siRNA targeting the PP2A catalytic unit for 48 hours and irradiated at 0, 2 and 4 Gy. Protein lysates were prepared after 48 hours post irradiation and were subjected to Western blot with an anti-LC3A/3B antibody. β-actin was used as a loading control. [file 1747-1028-8-10-S4.pdf]
